# How does social media use impact subjective well-being? Examining the suppressing role of Internet addiction and the moderating effect of digital skills

**DOI:** 10.3389/fpsyg.2023.1108692

**Published:** 2023-02-14

**Authors:** Bin Wu, Tianyuan Liu, Beihai Tian

**Affiliations:** ^1^Rural Construction & Management Research Center, Huazhong Agricultural University, Wuhan, China; ^2^School of Sociology, Wuhan University, Wuhan, China

**Keywords:** social media use, Internet addiction, subjective well-being, digital skills, flow theory

## Abstract

**Introduction:**

Previous studies have explored the impact of social media use on people’s subjective well-being, but there is a lack of discussion on the relationship between social media use, Internet addiction, and subjective well-being, and the research on the influence of digital skills on this relationship is not sufficient. This paper aims to fill these gaps. Based on the flow theory, this paper takes Chinese residents as the research object and uses CGSS 2017 data to analyze the impact of social media use on people’s subjective well-being.

**Methods:**

Our study used multiple linear regression models for analysis. To test the hypotheses and the moderated mediation model, we adopted PROCESS models with 5000 bias-corrected bootstrap samples and 95% confidence intervals. All analyses were conducted using SPSS 25.0.

**Results:**

The empirical analysis shows that social media use has a positive direct effect on subjective well-being, and Internet addiction plays a suppressing role in the relationship between social media use and subjective well-being. In addition, we found that digital skills moderated the positive effect of social media use on Internet addiction and the indirect effect of social media use on subjective well-being through Internet addiction.

**Discussion:**

The conclusion of this paper supports our previous hypothesis. Besides, the theoretical contribution, practical significance, and limitations of this study are discussed based on the results of previous studies.

## Introduction

With the changes of modern society, the Internet has penetrated into our daily life (Negroponte, 1995). In recent years, with the rise of social media applications and their growing number of users (Guo and Chen, 2022), more and more people are using social media as an important means of communication and entertainment. Social media are web-based channels through which people can present themselves and engage in continuous, unfettered interactions with familiar and unfamiliar people (Carr and Hayes, 2015). Users can also obtain the information they are interested in, browse short videos and shop online through social media, which brings satisfaction and convenience to them.

Based on these facts, the use of social media is generally believed to increase an individual’s subjective well-being (SWB). SWB is a state of overall satisfaction and happiness with life (Diener et al., 1997). It is also a self-judgment of life circumstances (Lifshitz et al., 2018). Numerous studies have shown that the use of social media has both direct and indirect positive effects on improving SWB. For example, research has found that entertainment-motivated social media use (SMU) can enhance self-disclosure (Kim et al., 2014). Image-based platforms such as Instagram and Snapchat provide a sense of intimacy and reduce users’ loneliness (Pittman and Reich, 2016), and reading or writing through social media can boost SWB (Yang, 2020). In addition, social media is also considered to satisfy people’s basic need to connect with others (Baumeister and Leary, 1995), thus promoting people’s social inclusion (Wei and Gao, 2017), which in turn enhances SWB (Pang, 2018).

However, some studies have reached the opposite conclusion, implying that SMU may have a negative impact on SWB (Shakya and Christakis, 2017). These studies suggest that SMU may exacerbate people’s social comparisons (Liu et al., 2019; Ozimek and Bierhoff, 2020), and problematic SMU may lead to boredom (Bai et al., 2021). Moreover, some scholars have pointed out that passive SMU weakens self-concept clarity (Lin et al., 2021), which further reduces SWB.

On either side of the divide, few studies have addressed the potential risk of Internet addiction associated with SMU. In particular, research on Internet addiction in China focuses on adolescents, students, and online games (Wang et al., 2013; Jiang, 2014; Wu et al., 2016). In practice, the newly revised *Law of the People’s Republic of China on the Protection of Minors* has added regulations to prevent minors from indulging in online games, and relevant game platforms have further upgraded their protection measures for minors to limit play and re-charging amounts. Face recognition and verification will be needed when minors log in to games and make payments (CNNIC, 2021). But there is less discussion on the relationship between SMU and Internet addiction in the general population (Li et al., 2021). In our opinion, all of these different effects should be included in the analysis. On the one hand, when people use social media, they can share their lives through writing, posting photos and short videos, and they can also interact with others through likes, comments, and private messages. These activities may bring positive feedback to users, making them feel happy and relaxed. On the other hand, people may also indulge in this way of obtaining happiness, resulting in Internet addiction and further adversely affecting their normal life and health. Further, how to preserve the positive impact of SMU on SWB as much as possible and reduce the risk of Internet addiction is less discussed.

In fact, China has experienced the rapid development of the Internet in the past few decades. According to the data of CNNIC (2021), as of June 2021, the number of netizens in China is 1.011 billion. Among netizens, 99.6% use mobile phones to access the Internet, and the Internet penetration in urban and rural areas reached 78.3% and 59.2%, respectively. The male-to-female ratio of netizens is 51.2:48.8, which is basically consistent with the male-to-female ratio of the overall population. In respect of age distribution, the percentage of netizens aged 30.39% was 20.3%, which was the highest among all age groups. The percentage of netizens aged 40–49 and 20–29 was 18.7% and 17.4% respectively, it ranks second and third in all age groups. In addition, under the joint efforts of the government, enterprises and society, the proportion of middle-aged and elderly netizens has increased significantly. By June 2021, netizens aged 50 or above accounted for 28.0%, an increase of 5.2% over June 2020. Moreover, in terms of different uses of the Internet, social media accounts for first place among Chinese netizens. With the rapid development of WeChat, QQ, and TikTok, social media has formed a huge user group among Chinese netizens. To be specific, the number of users of instant messaging social media reached 983 million, and the number of users of online video social media reached 944 million. The utilization rates of the two types of social media reached 97.3% and 93.4%. China’s large number of netizens and social media users provide sufficient conditions for our research. With social media nearly ubiquitous in China and happiness being the life goal of many people around the world (Tay et al., 2015), it is necessary to clarify how SMU affects people’s SWB directly, and whether SMU increases the risk of Internet addiction and reduces SWB.

This study is based on flow theory to explain the relationship between SMU and SWB in the context of Chinese society. Flow theory holds that when people devote their energy or “body and mind” to a certain activity, they may enter a state of flow, which in turn produces a high sense of excitement and fulfillment (Csikszentmihalyi, 2008). People may enter a state of flow when they are concentrating on using social media, which brings them a sense of pleasure and thus may boost their SWB. However, they may further maintain this state by increasing the frequency and duration of SMU, which exacerbates the risk of addiction (Csikszentmihalyi, 2008). Internet Addiction negatively affects their normal life and work, thereby lowering their SWB. Based on flow theory, we suggest that Internet addiction acts as a suppressor between SMU and SWB. Furthermore, digital skills may have moderated this relationship. According to the flow theory model, people need a balance between skill level and task difficulty to enter a flow state, and when people’s skill level exceeds the challenge they are dealing with, people will gradually feel bored and get out of this state (Csikszentmihalyi, 2000). Therefore, high skill levels may attenuate the relationship between SMU and Internet addiction, as well as the mediating effect of Internet addiction on SMU and SWB.

**Figure 1:**
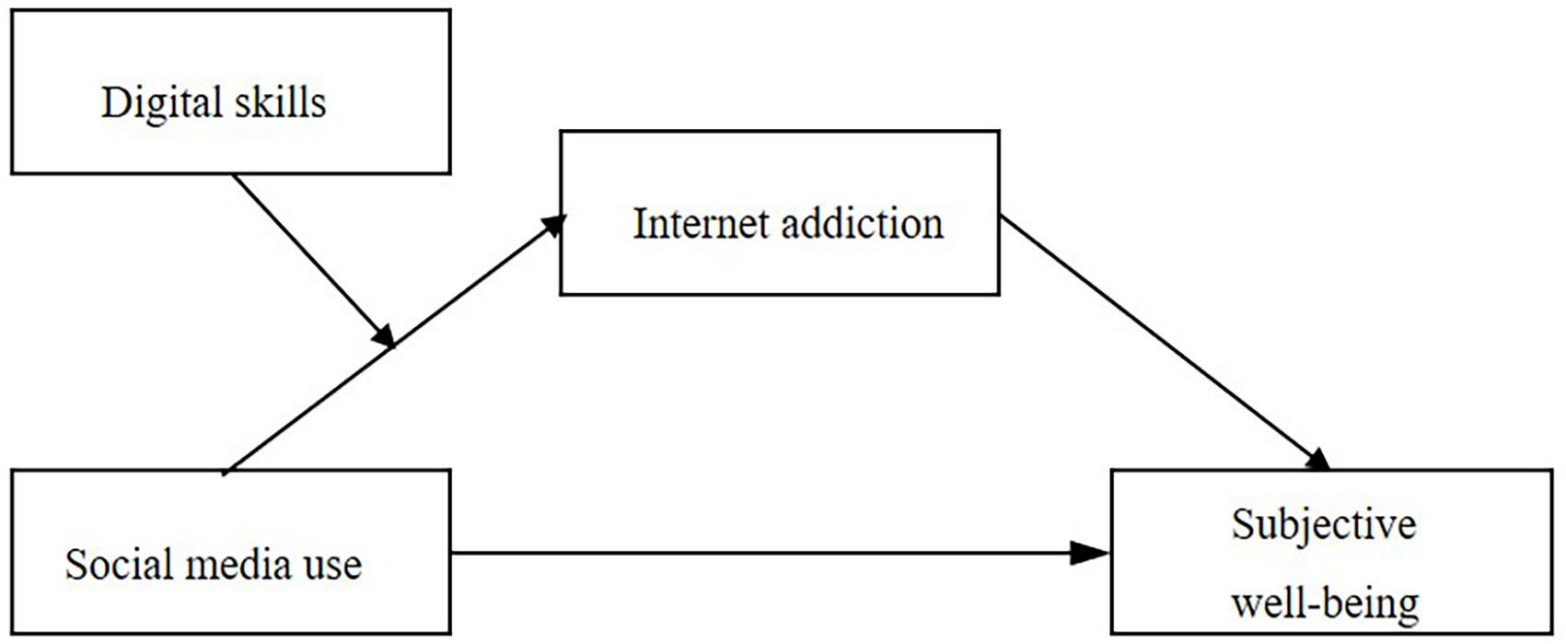
The conceptual mode of this research.

In summary, this study has two contributions to the SMU and SWB literature. First of all, there is little discussion on the relationship between SMU, Internet addiction, and SWB in existing research. To fill this gap, we apply the flow theory to demonstrate why SMU promotes SWB and how Internet addiction plays a suppressing role between SMU and SWB. Secondly, by testing the moderating mechanism of digital skills, the boundary conditions of SMU on Internet addiction and the mediating effect of Internet addiction on SMU and SWB were revealed. The conceptual model is shown in Figure 1.

## Literature review

### Direct effect of social media use on subjective well-being

SWB is people’s evaluation of life on the whole, which includes constructs such as happiness, life satisfaction, and morale (Kozma and Stones, 1980). Diener (1984) divided SWB into two types: cognitive SWB and affective SWB. The cognitive SWB includes life satisfaction, and the affective SWB includes the evaluation of emotional states such as happiness. People are considered to be at higher SWB levels when they have higher life satisfaction, more positive emotions, and less negative emotions (Diener et al., 1997). However, in the Chinese language culture, SWB is a general concept. People usually regard SWB as the overall feeling of happiness, which essentially includes satisfaction with life. In this study, we mainly focus on affective SWB, specifically, people’s overall evaluation of happiness.

Social media is a set of Web applications based on the ideas and technologies of Web 2.0. They allow people to create, share, and exchange content through their platforms (Kaplan and Haenlein, 2010). In the Chinese context, multiple types of social media serve different needs. For example, people can communicate with others through WeChat, share their lives on Weibo and WeChat Moments, acquire knowledge from Zhihu, relax by watching short videos through Tik Tok, and even go shopping by watching live broadcasts.

Flow theory suggests that when people focus on completing an activity or challenge to achieve some goals, they may enter a state of flow (Csikszentmihalyi, 2008), which makes them feel fulfilled, cognitively efficient, motivated, and happy (Moneta and Csikszentmihalyi, 1996). In other words, flow enhances people’s SWB. Three conditions must be met to enter a flow experience: (a) perceived challenge with a clear goal; (b) a balance between challenge difficulty and skill level; and (c) immediate feedback on progress (Csikszentmihalyi, 2000). The moderately challenging, enjoyable, and controllable nature of social media and its ability to provide immediate feedback to users make it possible to create an immersive experience for users, thus, social media has become an important source of flow experiences (Pelet et al., 2017) and beneficial to people’s SWB.

Along this line, when people use social media more frequently, they devote more of their attention to social media. While achieving their goals (such as chatting with friends on WeChat, and sharing their travel photos on Weibo), they may also receive positive feedback from their interactions with others. Through this flow experience, people gain relaxation and positive emotions, which directly improves their SWB (Kim et al., 2017). Therefore, we can make the following hypothesis:

*H1*: Social media use has a direct positive effect on subjective well-being.

### Suppressing effect of Internet addiction

Internet addiction is a broad term, and most scholars define it from the symptoms of addiction. Young (1998) believes that Internet addiction can be identified when an individual has 5 or more of the 8 symptoms. The 8 symptoms include: (1) addicted to the Internet; (2) need to spend more time online for gratification; (3) failed to quit Internet use; (4) reduced Internet use leads to negative emotions; (5) time spent online often exceeding expectations; (6) disrupting normal life due to the Internet; (7) concealing from others that one is overusing the Internet; (8) using the Internet as a way to escape problems and negative emotions. In general, when an individual is addicted to the Internet, surfing the internet will become his dominant activity, regulating his mood and further making him spend more time on the internet. This will bring him into conflict with the people around him, which will affect his work and life. In such a situation, preventing him from surfing the Internet will cause him to have negative emotions and adverse physical reactions, and it is easy to relapse even if the addiction is under control (Griffiths, 2000).

Flow theory states that when people are in a state of flow, they become so immersed in a stimulus that they lose the sense of time passing without noticing the potential harm. And they will repeatedly engage in activities that generate the flow experience to enjoy this happy state once again (Csikszentmihalyi, 2008), which greatly increases the likelihood of addiction.

When people use social media, the flow experience they get will increase their loyalty to these platforms (Zhou et al., 2010) and the frequency of SMU (Pelet et al., 2017), which boosts the risk of Internet addiction (Leung, 2014). Empirical research evidence suggests that internet addiction is detrimental to people’s mental health and that excessive internet use reduces people’s subjective well-being (Büchi et al., 2019; Guo et al., 2020; Balcerowska et al., 2022). In a word, the use of social media may increase the risk of Internet addiction, and thus reduce people’s SWB. The reasoning above suggests that SMU benefits people’s SWB, so Internet addiction may suppress the positive effects of SMU. This leads us to hypothesis 2:

*H2*: Internet addiction suppresses the positive relationship between social media use and subjective well-being.

### Moderating effect of digital skills

Digital skills are one of the prerequisites for using social media. van Laar et al. (2017) and other scholars believe that the core digital skills in the 21st century should include a total of seven aspects: technical, information management, communication, collaboration, creativity, critical thinking, and problem solving. It can be considered that digital skills not only include basic skills such as installation, opening, and browsing applications but also include advanced skills such as using digital tools to collect information, identify information, release information, deal with affairs, and communicate with others. Therefore, the level of digital skills will affect the breadth and depth of a user’s SMU.

According to the flow theory model, people’s flow state is a kind of dynamic balance that requires matching their skill level with the difficulty of the task. When people’s skill level exceeds the difficulty of the task, this balance will be upset and people will start to feel bored, while when people’s skill level cannot meet the requirements of the task, people will feel anxious (Csikszentmihalyi, 2000).

In this study, the skills people needed to achieve a flow experience were digital skills, and the task was to get pleasure and satisfaction from using social media to achieve goals. According to flow theory (Csikszentmihalyi, 2000), achieving a state of flow through SMU requires a balance between digital skills and the difficulty of the task. As a mass media with a very wide audience, social media has relatively low barriers to use and technical difficulty.

As a result, even low levels of digital skills are compatible with the difficulty of using social media, making it easier to have a flow experience and thus more likely to become addicted to it. However, people with high digital skills, whose skill level is higher than the difficulty of using social media, may feel bored soon after they enter the state of flow and choose to stop using social media or pursue a more difficult way of using it, so they are not easy to indulge in it (Moneta and Csikszentmihalyi, 1996). Thus, we suggest that digital skills may moderate the relationship between SMU and Internet addiction, and people with higher levels of digital skills may have a lower risk of Internet addiction. We propose the following assumptions:

*H3*: Digital skills moderates the relationship between social media use and Internet addiction. Specifically, this relationship will be weakened when people have higher digital skills.

In addition, based on the above hypothesis, when people’s digital skill level is higher, the positive effect of SMU on Internet addiction is weaker, while Internet addiction is negatively correlated with SWB. Accordingly, it can be speculated that when people’s digital skill level is higher, the negative indirect effect of SMU on SWB through Internet addiction is weaker. Consequently, we propose the following hypothesis:

*H4*: Digital skills moderates the indirect effect of social media use on subjective well-being via Internet addiction. Specifically, the indirect effect will be weakened when people have higher digital skills.

## Materials and methods

### Sampling

The data for this study come from the Chinese General Social Survey 2017 (CGSS 2017). CGSS was founded and conducted by RUC (Renmin University of China), one of China’s top universities, and funded by the “985” Foundation and the Scientific Research Foundation and implemented by the China Survey and Data Center of RUC. Since 2003, it has conducted a continuous cross-sectional survey of more than 10,000 households in all provinces and autonomous regions of the Chinese mainland once a year. CGSS systematically and comprehensively collects data at multiple levels of society, community, family and individual, summarizes the trend of social change, discusses issues of great scientific and practical significance, provides data for international comparative research, and acts as a multidisciplinary economic and social data collection platform. At present, CGSS has become the most important data source for the study of Chinese society, which is widely used in scientific research, teaching, and government decision-making. It is the earliest national, comprehensive, and continuous academic survey project in China.

In terms of sampling design, CGSS adopts multi-stage stratified PPS random sampling, which is divided into three sampling stages. In the first stage, the sampling unit is county, and a total of 100 counties are selected; in the second stage, the sampling unit is community, 4 communities are selected in each county; in the third stage, the sampling unit is household, 25 households are selected in each community. The questionnaire was collected by face-to-face interview. The interviewees will receive a letter of introduction and a small gift when the investigator enters their homes, and will participate in the survey on a voluntary basis. CGSS strictly adheres to the ethics of scientific research and Chinese laws and keeps all personal information provided by the interviewees confidential.

As for research topics, CGSS 2017 is divided into three modules: core module, social network and Internet society module, and family questionnaire module. The core module collected demographic information, socio-economic information, such as education level, family income, occupation information, and social attitude. The social network and Internet society module collected information about respondents’ social networks, social interactions, social participation, and Internet use. The family questionnaire module collected information about the respondents’ family structure information, old-age support, the family values and so on. It is particularly worth mentioning that CGSS 2017 data contains questions about respondents’ use of the Internet, which is a rare and nationally representative data on individual Internet use in China. The data of this study contains 783 variables. After data cleaning and variable screening, a total of 2,137 samples were finally included in this study.

### Measurement

The complete measurement items and reliability and validity test results of the main research variables are shown in Table 1.

**Table 1:** Reliability and validity test of the scale and confirmatory factor analysis properties.

Construct	Factor loadings	CR	AVE
**Digital skills**			
I can use the computer to open the website	0.777^***^	0.901	0.606
I can download and install apps using my smartphone	0.839^***^		
It is not hard to find the information you want on the Internet	0.868^***^		
When I see important news forwarded by people around me on the Internet (such as WeChat and Weibo), I will verify it before I believe it	0.646^***^		
When I want to express myself online, I know how to do it	0.799^***^		
When making payments or transactions online, I will observe the usage environment to determine whether to use	0.719^***^		
**Internet addiction**			
**Symptoms of addiction**			
I spend more time online than before	0.689^***^	0.790	0.487
When I am in a bad mood, I surf the Internet so that I feel better	0.690^***^		
I often stay online longer than I planned	0.782^***^		
I get restless if I do not surf the Internet for a while	0.620^***^		
**Interpersonal and daily life problems**			
Because of the Internet, my daily life has been affected	0.824^***^	0.800	0.506
Because of the Internet, my work has been affected	0.786^***^		
Because of the Internet, I have become more estranged from the people around me	0.619^***^		
My family complains that I spend too much time online	0.585^***^		
**Health problems**			
Because of the internet, I spend less and less time going out	0.710^***^	0.738	0.485
Because of the internet, my eyesight has deteriorated	0.691^***^		
Because of the internet, my shoulders and cervical spine hurt	0.687^***^		

Standardized coefficients reported; ^***^p < 0.01; AVE, Average variance extracted; CR, Composite reliability.

#### Subjective well-being

As described above, this study focuses on affective subjective well-being (SWB), which mainly refers to the level of happiness in the current life. To measure SWB, previous researchers have developed many scales, such as Satisfaction with Life Scale (SWLS; Diener et al., 1985) and Scale of Positive and Negative Experience (SPANE; Diener et al., 2010). Some scholars also use a single item to measure SWB, such as by asking respondents to rate their overall happiness (0 = completely unhappy, 10 = completely happy) to measure their SWB (Arampatzi et al., 2018). CGSS referenced this measurement and redesigned the options to a 5-scale scale. Specifically, the SWB was measured in CGSS by answers to the question “In general, how happy would you say your current life is?” (Liu et al., 2020; Ding et al., 2021). There were 5 options for this question, and they were assigned a value from 1 to 5, namely “very unhappy,” “unhappy,” “neither happy nor unhappy,” “happy” and “very happy.” These scores were used to measure respondents’ subjective well-being (M = 3.910, SD = 0.802).

#### Social media use

Respondents were asked how often they had engaged in a range of online activities in the past year, including communication, self-presentation, online rights protection, entertainment, gaining information, and online transactions. Each item has 5 options, and the values are 1 to 5 (1 = “never”; 5 = “always”). These scores were added and averaged, which were used to measure the social media use (SMU; M = 3.021, SD = 0.782).

#### Internet addiction

Respondents were given a list of 11 statements to rate their level of agreement on a 1–5 scale (1 = “strongly disagree” to 5 = “strongly agree”) in CGSS 2017. These statements are based on the Chinese Internet Addiction Scale (Chen et al., 2003) and include some symptoms of Internet addiction and its negative effects, such as “I spend more time online than before” and “Because of the internet, my eyesight has deteriorated.” The scale contains three dimensions, namely “Symptoms of addiction,” “Interpersonal and daily life problems” and “Health problems.” The scores were averaged to form a measure of Internet addiction (M = 2.668, SD = 0.749). The reliability for the scale as indicated by Cronbach’s a was remarkably high at 0.872.

#### Digital skills

Some scholars believe that digital skills include six core dimensions, namely technical, information management, communication, collaboration, creativity, and critical thinking (van Laar et al., 2017). CGSS partly refers to the division of this dimension and designs the Digital Skills Scale according to the Chinese context. The scale consists of six items such as “I can use the computer to open the website” and “When I want to express myself online, I know how to do it.” The items cover basic digital skills, information collection, information identification, information creation and self-expression, and information security. Each item has 5 options, and the values are 1 to 5 (1 = “strongly disagree”; 5 = “strongly agree”). The scores were averaged to measure the level of digital skills (M = 3.882, SD = 1.022). Cronbach’s alpha for this scale was 0.900.

#### Control variables

Control variables included 4 sets of variables: demographic information, subjective socioeconomic status, perceived health status, perceived social equality, and perceived social trust. The demographic information included gender (0 = “female”; 1 = “male”), age(year), education level (1 = “elementary”; 2 = “intermediate”; 3 = “advanced”), marriage status (0 = “single/widowed/divorced”; 1 = “married”), hukou (0 = “rural hukou”; 1 = “urban hukou”), and current residence (0 = “rural area”; 1 = “urban area”). Subjective socioeconomic status was measured by asking “Taken together, which level of society are you currently at?.” The answers ranged from 1 (the bottom) to 10 (the top). Perceived health status was measured by respondents’ self-rated health scores (1 = “unhealthy”; 5 = “very healthy”). Perceived social equality was measured by asking respondents about their overall perceptions of equality and trust in today’s society (1 = “totally unequal”; 5 = “completely equal”). Moreover, respondents were asked “In general, most people in this society can be trusted?” to assess their perceived social trust (1 = “strongly disagree”; 5 = “strongly agree”).

### Data analysis

In our study, SWB, SMU, digital skills, and Internet addiction were considered as continuous variables, so we used multiple linear regression models for analysis. To enhance the robustness of regression results, all control variables are included in the model. To test the hypotheses and the moderated mediation model, we adopted PROCESS models with 5,000 bias-corrected bootstrap samples and 95% confidence intervals. If the effect does not include 0 in the 95% confidence interval, it is statistically significant. All analyses were conducted using SPSS 25.0.

## Results

### Survey reliability, validity, and common method bias testing

Before testing the research hypotheses, we first tested the reliability and validity of the two scales and the common method bias of this study. For survey reliability, we used composite reliability (CR) to measure the reliability of the two scales in the study. The results in Table 1 showed that the composite reliability of the four factors all exceeded the recommended value of 0.7. Therefore, the reliability of the scale used in this study is high.

Regarding survey validity, we performed confirmatory factor analysis to calculate standardized factor loadings for each item in the two scales. The Bartlett sphericity test statistic values of the two scales were significant at the 0.1% level, and the KMO values were all greater than 0.7, which were suitable for factor analysis. The standardized factor loadings of all items of the two scales ranged from 0.585 to 0.868, which were all greater than the threshold of 0.5 (Hair et al., 2014). In addition, the AVE (average variance extracted) value of the Digital Skills Scale exceeded 0.5, while two of the three AVE values of the Internet Addiction Scale were close to 0.5 and one exceeded 0.5, indicating that this study has relatively good discriminant validity.

To reduce the common method bias, the data collection process of CGSS followed strict procedures, and a large number of reverse scoring items were designed. We also adopted Harman’s single-factor analysis using exploratory factor analysis (Podsakoff et al., 2003). The results showed that the single factor explained 22.352% of the variance, which is less than the 40% standard proposed. Therefore, it can be considered that there is no serious problem of common method bias in this study. SPSS 25.0 and Mplus 8.0 were used to calculate all the indicators above.

### Descriptive statistics

Table 2 shows the descriptive statistical results and correlations of the core variables of this study, including the means, standard deviations, and correlation coefficients. There were close relationships between the core variables in the study. For example, SWB was positively correlated with SMU (*r* = 0.082, *p* < 0.01) and digital skills (*r* = 0.118, *p* < 0.01), and negatively correlated with Internet addiction (*r* = −0.056, *p* < 0.01). As expected, SMU was significantly associated with Internet addiction (*r* = 0.451, *p* < 0.01). In addition, digital skills was positively correlated with SMU (*r* = 0.679, *p* < 0.01).

**Table 2:** Results of the correlation analysis.

	SWB	SMU	Digital skills	Internet addiction
SWB	1.000			
SMU	0.082^***^	1.000		
Digital skills	0.118^***^	0.679^***^	1.000	
Internet addiction	−0.056^***^	0.451^***^	0.378^***^	1.000
Mean	3.910	3.021	3.882	2.668
SD	0.802	0.782	1.022	0.749

^*^p < 0.1; ^**^p < 0.05; ^***^p < 0.01; SWB, Subjective well-being, SMU, Social media use.

### Hypothesis testing

Hypothesis 1 of this study posits that SMU directly improves SWB. The regression results in Table 3 showed that SMU has a positive impact on SWB (ß = 0.055, *p* < 0.05; Model 3), supporting Hypothesis 1.

**Table 3:** Results of the mediation model and moderation model regression analysis.

	Model 1	Model 2	Model 3
	S	S	SWB
**Predictor**			
Social media use (IV)	0.363^***^	0.593^***^	0.055^**^
	(0.022)	(0.069)	(0.026)
**Suppressor**			
Internet addiction(S)			−0.071^***^
			(0.024)
**Moderator**			
Digital skills(W)		0.257^***^	
		(0.048)	
**Interaction**			
IV*W		−0.073^***^	
		(0.017)	
**Control variables**			
Age	−0.008^***^	−0.007^***^	0.003^*^
	(0.001)	(0.001)	(0.002)
Gender	0.012	0.004	−0.042
	(0.029)	(0.029)	(0.032)
Education level	0.034	0.023	0.078^**^
	(0.028)	(0.029)	(0.031)
Marriage status	−0.108^***^	−0.127^***^	0.217^***^
	(0.037)	(0.037)	(0.041)
Hukou	−0.007	−0.020	0.024
	(0.037)	(0.037)	(0.041)
Current residence	−0.015	−0.019	−0.017
	(0.040)	(0.039)	(0.043)
Subjective socioeconomic status	0.000	−0.002	0.064^***^
	(0.009)	(0.009)	(0.010)
Perceived health status	−0.055^***^	−0.059^***^	0.175^***^
	(0.016)	(0.016)	(0.018)
Perceived social equality	−0.015	−0.016	0.139^***^
	(0.015)	(0.015)	(0.016)
Perceived social trust	−0.024	−0.024^*^	0.096^***^
	(0.015)	(0.014)	(0.016)
Constant	2.256^***^	1.493^***^	1.817^***^
	(0.128)	(0.199)	(0.151)
N	2,137	2,137	2,137
R^2^	0.234	0.245	0.195

^*^p < 0.1; ^**^p < 0.05; ^***^p < 0.01; Cell entries are unstandardized coefficient with standard errors in parentheses; SWB, Subjective well-being.

Hypothesis 2 assumes that Internet addiction will suppress the positive impact of SMU on SWB. First, the control variables and the independent variables were entered into Model 1. The regression result of Model 1 indicated that SMU had a positive relationship to Internet addiction (ß = 0.363, *p* < 0.01; Model 1). Second, Model 3 included Internet addiction, SMU, and all control variables. Regression results represented that Internet addiction is not conducive to SWB (ß = −0.071, *p* < 0.01; Model 3). In the mediation model, when the coefficients of the direct and indirect effects have opposite signs, it is a suppressing effect (Tzelgov and Henik, 1991). Therefore, we believe that Internet addiction attenuates the positive effect of SMU on SWB as a suppressor. Hypothesis 2 was supported.

**Figure 2:**
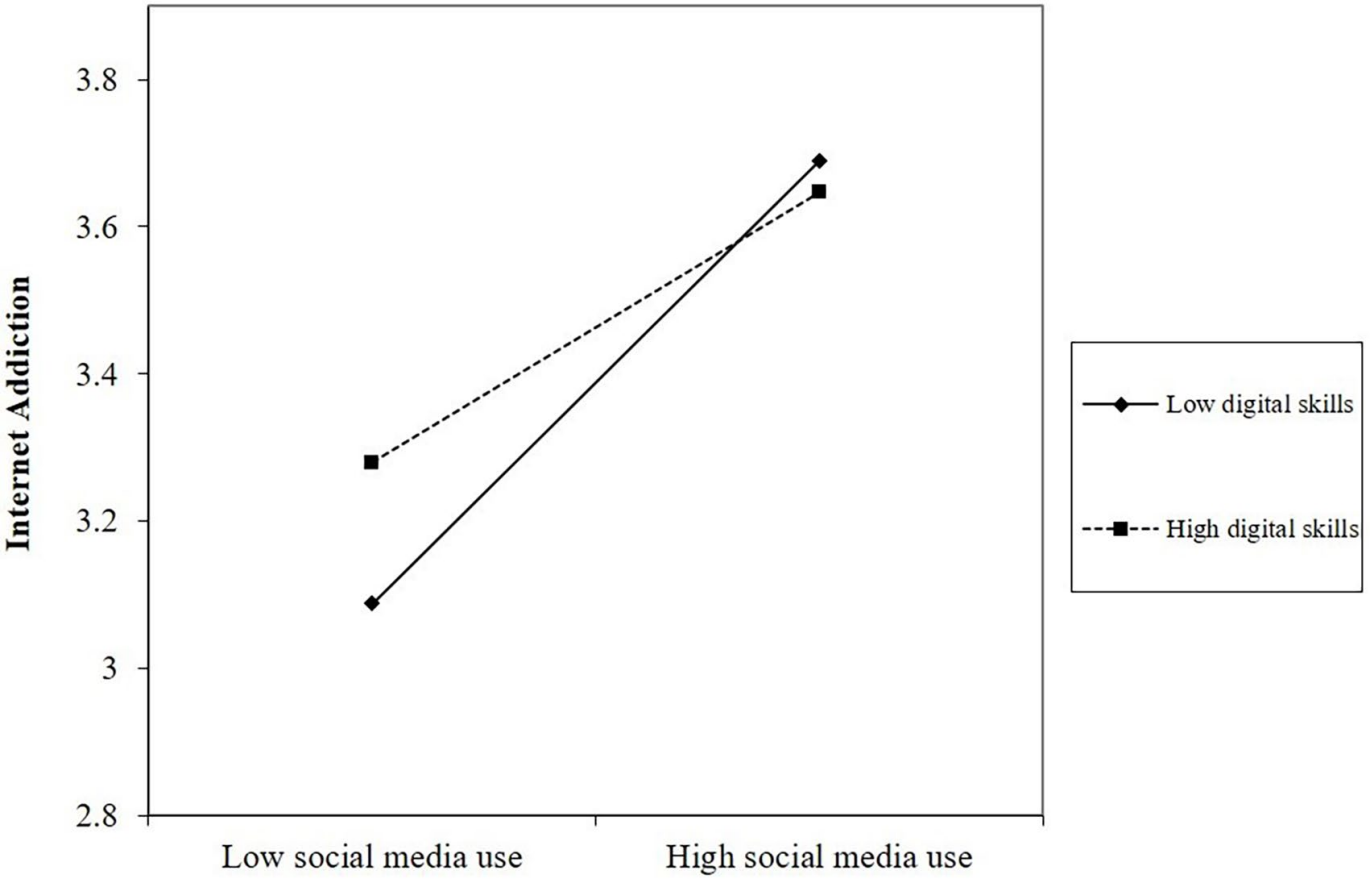
The moderation effect of digital skills on the relationship between SMU and Internet addiction.

For Hypothesis 3, the moderated causal step approach was adopted for testing. Hypothesis 3 assumes that digital skills moderates the relationship between SMU and Internet addiction. As Table 3 showed, the interaction significantly and negatively affected Internet addiction (ß = −0.073, *p* < 0.01; Model2), supporting Hypothesis 3. This indicates that under the condition of a certain level of SMU, people with higher digital skills are less likely to become addicted to the Internet. We then calculated the slope of digital skills at low (Mean − 1SD) and high levels (Mean + 1SD) and plotted the moderation patterns. As shown in Figure 2, when individuals had a low level of digital skills, SMU exerted a stronger positive influence on Internet addiction than the individuals who had a high level of digital skills. Hypothesis 3 was verified again.

In addition, bootstrap sampling was performed 5,000 times to further examine the direct effect, suppressing effect, and moderating effect. If the 95% confidence interval does not contain zero, the effect is significant. The analysis results in Table 4 showed that SMU had a significant direct effect on SWB (ß = 0.055, Boot SE = 0.026, CI = [0.004, 0.105], CI did not contain 0), which again confirmed Hypothesis 1. Besides, the path coefficient of SMU affecting SWB *via* Internet addiction was significantly negative (ß = −0.026, Boot SE = 0.009, CI = [−0.044, −0.008]) and the confidence interval did not include 0, which verified Hypothesis 2 again. At last, the results in Table 5 also showed the indirect effect of SMU on SWB *via* Internet addiction was significant and negative, regardless digital skills was high or low, and the absolute value was the lowest when digital skills was at high level (ß = −0.017, Boot SE = 0.006, CI [−0.031, −0.005]). The index of moderated mediation was also significant (ß = 0.005, Boot SE = 0.002, CI [0.001, 0.010], CI did not contain 0). Hence, these findings provided support for Hypothesis 4.

**Table 4:** Non-standardized mediation analysis results.

Paths	Effect	Boot SE	LLCI	ULCI
**Direct effect**				
IV → DV	0.055	0.026	0.004	0.105
**Indirect effect**				
IV → Internet addiction→DV	−0.026	0.009	−0.044	−0.008

IV, Social media use; DV, Subjective well-being; Bootstrap resample = 5,000; Control variables are included in the analysis.

**Table 5:** Moderated mediation results.

Paths	Effect	Boot SE			LLCI	ULCI
**IV → Internet addiction → DV**						
Digital skills = Mean-1SD			−0.027	0.010	−0.047	−0.008
Digital skills = Mean			−0.022	0.008	−0.038	−0.007
Digital skills = Mean + 1SD			−0.017	0.006	−0.031	−0.005
Index of moderated mediation			0.005	0.002	0.001	0.010

IV, Social media use; DV, Subjective well-being; Bootstrap resample = 5,000; Control variables are included in the analysis.

## Discussion

### Summary of findings

This study mainly explored the direct effect of SMU on people’s SWB, and the specific mechanism. Data analysis of 2,137 Chinese residents shows that SMU has a positive direct effect on SWB, and SMU has a negative indirect effect on SWB through Internet addiction. Furthermore, people with low digital skills are more likely to become Internet addicts through SMU than those with high digital skills. In addition, the level of digital skills also moderated the indirect effect of SMU on SWB through Internet addiction. Our research also has some theoretical and practical implications, which we will discuss in the next section.

### Theoretical implications

The use of social media is becoming more and more common today. Scholars have done a lot of research on the influence of SMU, especially on SWB. However, few studies have considered Internet addiction as a possible mechanism between SMU and SWB. Also, there has been insufficient discussion of the impact of digital skills on this mechanism. The role of digital skills in reducing the likelihood of Internet addiction should be emphasized. To fill these research gaps, we take them as important research questions and conduct empirical analysis in the Chinese context. Therefore, the results of this study have theoretical significance.

First, this study explores the direct effect of SMU on SWB. Although many empirical studies have demonstrated the positive effects of SMU on SWB, some studies have proved that SMU is not conducive to SWB. To examine the role of SMU on SWB, this study introduced Internet addiction as a suppressor into the analysis model and included all control variables. The results showed that SMU had a positive direct effect on SWB, which was in agreement with some researches (Kim et al., 2014; Pittman and Reich, 2016; Yang, 2020). Therefore, the results further confirmed the positive direct impact of SMU on people’s SWB.

Secondly, according to the flow theory, the important mechanism of SMU’s influence on people’s SWB is revealed. Specifically, SMU lowered people’s SWB through the suppressing effect of Internet addiction. At the same time, SMU will increase people’s risk of Internet addiction, which means that SMU has a positive impact on people, but also increased the potential harm. Therefore, our research also contributes to the study of Internet addiction.

Finally, based on the flow theory model, this study demonstrates the role of digital skills in moderating the relationship between SMU and SWB. Specifically, people with higher levels of digital skills have less difficulty using social media and get bored soon after the flow experience. Therefore, they are not easy to indulge in SMU and give full play to the positive direct effect of SMU on SWB. In contrast, for people with lower levels of digital skills, SMU matches their skill levels and is therefore prone to a sustained flow experience, which increases the risk of Internet addiction and further reduces their SWB. In conclusion, the results confirm the boundary condition of SMU leading to Internet addiction, and further expand the research on SMU.

### Practical implications

Given the positive direct effects of SMU on SWB, the suppressing effect of Internet addiction, and the moderating effect of digital skills, this study has some practical implications.

On the one hand, the government and relevant departments should continue to guide the public to use social media moderately, so that social media can play a positive role and improve people’s SWB. Relevant companies should make social media more practical, interesting, and easy to use so that more people can benefit from it. For example, develop new functions to attract more user groups, and add children mode and elderly mode to facilitate different groups.

On the other hand, digital skills are essential for the survival of the public today. The government, schools, and enterprises should focus on improving the public’s digital skills to reduce the risk of Internet addiction caused by SMU and lower its negative impact on SWB. To be specific, people should be helped to learn basic digital skills, while enhancing their ability to use social media to manage information, create information, express themselves and facilitate their lives, as well as promote information security awareness. In addition, we also need to guide people to use social media moderately to prevent Internet addiction and reduce SWB. Enterprises and departments also need to step up the development of anti-addiction systems to help people avoid excessive use of social media.

### Limitations and future directions

Although our research has some theoretical and practical significance, it still has some limitations. First of all, the sample of this study is from CGSS based on the Chinese social context, and people’s perception of SWB and specific SMU may be different from other countries. More studies of different countries are needed in the future to facilitate cross-cultural comparisons. Also, we did not segment respondents by age group. Does the relationship between SMU and SWB differ among different age groups? Are the underlying mechanisms consistent? Meanwhile, in the context of the COVID pandemic in recent years, are there any new changes in the role of social media and its impact on people’s SWB? These require further exploration.

Secondly, this study integrates multiple social media use activities and examines the impact of SMU on internet addiction and SWB. However, different types of SMU may have different outcomes. In future research, we can classify and compare the risk of internet addiction brought by different types of SMU and their effects on SWB.

Third, limited by the research design of CGSS, we were able to obtain relevant variables of SMU only in the CGSS 2017 data and not in the data for subsequent years. While CGSS asked respondents about their SMU in the past year, it asked about their current feelings when collecting information about SWB. Therefore, there is a time lag between the two variables, which partly explains why the SMU is the cause and the SWB is the result. Nevertheless, causality in our study still needs to be further validated with longitudinal study data or more rigorous experimental design, which is also an important work to be done in future studies.

Finally, restricted to the questionnaire design, SWB is measured using the respondents’ overall assessment of their current life, which may not provide more comprehensive information. Future studies should consider the differences between different SWB dimensions when studying the relationship between SMU and SWB.

## Conclusion

Previous studies have not sufficiently discussed the relationship between SMU, Internet addiction and SWB, and the possible influencing mechanism of digital skills in this relationship is still unclear. Based on the Chinese context, this study explores the relationship between SMU, Internet addiction, and SWB. The results of empirical analysis confirmed that SMU had a positive direct effect on SWB, and Internet addiction as a suppressor weakened the positive effect of SMU on SWB. Furthermore, digital skills is an important moderating factor, negatively moderating the indirect effects of SMU and SWB. Also, the findings of our research provide a perspective to understand the benefits of SMU for SWB and the potential risk of Internet addiction, which can help relevant authorities recognize the important role of digital skills and reduce the adverse effects through relevant measures.

## Data availability statement

The original contributions presented in the study are included in the article/Supplementary material, further inquiries can be directed to the corresponding author.

## Author contributions

BW contributed in writing the original draft, conceptualization, data curation, formal analysis, and methodology. BT contributed in editing and supervision of the paper. TL contributed in data curation, methodology, review, and editing. All authors contributed to the article and approved the submitted version.

## Funding

This study was funded by The Fundamental Research Funds for the Central Universities of China (grant number 2662020WFPY003) and The National Social Science Fund of China (grant number 22CSH035).

## Conflict of interest

The authors declare that the research was conducted in the absence of any commercial or financial relationships that could be construed as a potential conflict of interest.

## Publisher’s note

All claims expressed in this article are solely those of the authors and do not necessarily represent those of their affiliated organizations, or those of the publisher, the editors and the reviewers. Any product that may be evaluated in this article, or claim that may be made by its manufacturer, is not guaranteed or endorsed by the publisher.
